# PERSPECTIVES OF OLDER RURAL CANCER SURVIVORS ON REMOTELY DELIVERED ENHANCE FITNESS

**DOI:** 10.1093/geroni/igae098.0997

**Published:** 2024-12-31

**Authors:** Nancy Gell, Jacqueline Caefer, Kushang Patel

**Affiliations:** University of Vermont, Burlington, Vermont, United States; University of Vermont, Burlington, Vermont, United States; University of Washington, Seattle, Washington, United States

## Abstract

Exercise is recommended for healthy aging and to counteract long term effects of cancer and cancer treatment. Older cancer survivors living in rural communities face unique challenges to accessing exercise opportunities including fewer in-person program options, transportation barriers, and lack of safe, outdoor infrastructure. Remotely delivered exercise provides opportunities to overcome many of the documented barriers to exercise for this underserved population. The purpose of this study was to examine perspectives from older rural cancer survivors after participating in remotely delivered Enhance Fitness group exercise (Tele-EF). We conducted semi-structured interviews with 27 older cancer survivors living in rural communities (mean age 70.8 ±5.7, 81% female) after they completed 16-weeks of Tele-EF. All interviews were audio-recorded and transcribed. Transcripts from the interviews were coded using inductive thematic analysis. Analysis and discussion of the codes resulted in iterative mapping of key themes to the Capability Opportunity and Motivation Behavioral (COM-B) framework. Emergent themes related to Tele-EF included: 1. Capability: Improvements in psychological and physical capacity to exercise resulted from supportive instruction, experiential learning, and exercise modifications by trained instructors. 2. Opportunity: Barriers of transportation and weather were eliminated while technology, when functioning, afforded accessibility at home; comradery prompted consistent attendance. 3. Motivation for exercise was enhanced through improved capability, opportunity, and functional improvement. Tele-EF facilitates exercise engagement for older rural cancer survivors through constructs identified in the COM-B framework. Future work should examine how to optimize technology for engaging older rural cancer survivors in synchronous, remote exercise.

